# Changes in the nutrients, phytochemical profile and antioxidant activity of *Rheum officinale* Baill. leaf blades during different growth periods

**DOI:** 10.3389/fnut.2024.1387947

**Published:** 2024-04-17

**Authors:** Lixia Dai, Xiaolou Miao, Yudong Ma, Xiaorong Yang, Bing Li, Jian He, Yu Wang, Pengcheng Dong, Jiyu Zhang, Xiaofei Shang

**Affiliations:** ^1^College of Veterinary Medicine, Gansu Agricultural University, Lanzhou, China; ^2^Key Laboratory of New Animal Drug Project, Key Laboratory of Veterinary Pharmaceutical Development of Ministry of Agriculture, Lanzhou Institute of Husbandry and Pharmaceutical Sciences, Chinese Academy of Agricultural Sciences, Lanzhou, China; ^3^Lanzhou Jiaotong University, Lanzhou, China; ^4^Gansu Herbage and Livestock Environment Observation and Research Station, Lanzhou Institute of Husbandry and Pharmaceutical Sciences, Chinese Academy of Agricultural Sciences, Lanzhou, China

**Keywords:** *Rheum officinale*, chemical composition, nutrient value, harvest season, antioxidant activity

## Abstract

Rhubarb contains an abundance of compounds and nutrients that promote health through various activities; however, these activities are affected by the harvest season. In this paper, the changes in nutrients, phytochemical profiles and antioxidant activity of *Rheum officinale* leaf blades (LRO) during different growth periods were investigated. The results showed that LRO is a good source of protein, fiber, and minerals and contains abundant fatty acids; however, as the harvest time increased from March to July, the levels of protein and amino acid decreased, and the levels of other nutrients reached a maximum in May or June. LRO also contains flavonoids, terpenoids, and quinones. As the harvest time increased, the quinone content decreased, possibly due to the unstable chemical properties of quinones at high temperatures. The flavonoid contents reached a maximum in May or June. This study indicated that LRO is a source of nutrients and chemical components and can be used for functional food production. In addition, the nutrients and chemical components related to the antioxidant activity of LRO changed according to the harvest season.

## 1 Introduction

The genus *Rheum* (Polygonaceae) is widely distributed and cultivated worldwide (1, 2). Among the approximately 50 species in this genus, the dried rhizomes and roots of *R. palmatum*, *R. tanguticum*, and *R. officinale*, which exhibit purgative, antibacterial and antifungal, anti-inflammatory, antitumor, and hepatoprotective activities (3, 4), were identified as official rhubarbs and are listed in the United States Pharmacopeia, Chinese Pharmacopeia, European Pharmacopeia, and Pharmacopeia in other Asian countries (5–7).

As an important herb in this genus, *R. officinale* is widely distributed in some Asian countries. In China, this species is cultivated in northwestern regions, such as Gansu, Qinghai, Inner Mongolia and Sichuan provinces (3). Approximately 5,500 tons of dried rhizomes and roots are produced annually (8), and these products are used in folk medicine to treat diarrhea, constipation, abdominal pain and jaundice. The fresh aerial parts, including stalks, stems and leaves, produce a sour taste and are eaten as vegetables by local people and animals in some regions; however, many raw materials are discarded. To further develop the production and use of the aerial parts as a functional food, we previously performed investigations and revealed that *R. officinale* stalks exhibit good safety; contain an abundance of essential amino acids, essential fatty acids, fiber, and minerals; and can be used as sources of nutrients in the food industry to regulate the body’s immunity (3). Further study revealed that more nutrients and chemical components are present in the leaves than stalks, which contain abundant pectin that can be used as dietary fiber (9). However, the nutrients in *R. officinale* leaves have not been comprehensively investigated, and the phytochemical profile and antioxidant activity of these components have not been elucidated.

The nutrient content and phytochemical profile of plants are influenced by several factors, including different growth periods, soils, cultivation techniques, and climatic conditions (10). As the leaf blades are harvested in March, it is appropriate to investigate the effect of the harvest period on the content of biologically active components (11). In this paper, the potential nutrients found within the leaves during the different growth periods, including minerals, amino acids, and fatty acids, were identified. In addition, the phytochemical profile and antioxidant activities of *R. officinale* leaf blades (LRO), which were harvested during different growth periods from March to July, were evaluated. This paper lays the foundation for harvesting LRO in a suitable season for further application in the food industry.

## 2 Materials and methods

### 2.1 Plant material

Leaf blades of *R. officinale* (LRO) were harvested during March–July from Kang County (33.00’ S, 105.77′ E) in the Longnan region of Gansu Province, China. The samples were identified by Prof. Chaoying Luo from the Lanzhou Institute of Husbandry and Pharmaceutical Sciences, CAAS (Lanzhou, China). Then, the fresh LRO was dried to constant weight in an oven at 60°C for further use, after which the moisture content was determined.

### 2.2 Reagents

Sodium acetate, sodium citrate buffer and ascorbic acid (Vc) were purchased from Sinopharm Chemical Reagent Co (Shanghai, China). Methanol and acetonitrile (MS and HPLC grade) were purchased from Fisher Scientific (England). 2,2-Diphenylpicrylhydrazyl (DPPH), nicotinamide adenine dinucleotide disodium salt (NADH-2Na), ammonium acetate (NH_4_AC), ammonium hydroxide (NH_4_OH), undecanoic acid, amino acid standards (19 L-amino acids, including Ala, Gly, Val, Leu, Ile, Phe, Met, Pro, Ser, Thr, Cys, Tyr, Asn, Gln, His, Lys, Arg, Asp., Glu), and minerals (including calcium (Ca), copper (Cu), iron (Fe), potassium (K), sodium (Na), magnesium (Mg), manganese (Mn), phosphorus (P), selenium (Se), and zinc (Zn)), and Folin–Ciocalteu reagent were obtained from Sigma–Aldrich (Shanghai, China). Hyperoside (98%), emodin (98%), rhein (98%), aloe-emodin (98%), myricitrin (98%), kaempferol (98%), isoorientin (98%), and gallic acid (98%) were purchased from Yuanye Co. (Shang, China) and Macklin Co. (Shang, China), respectively.

### 2.3 Nutrients

#### 2.3.1 Proximate compositions

The crude lipid (CL), total protein (TP), total dietary fiber (DF), ash (AS), and digestible carbohydrate (DC) contents in LRO were analyzed according to a previously described method (10). The levels of crude lipids and dietary fibers were determined using a Soxtec 2050 Automatic Soxhlet Extraction Analyzer (FOSS, Denmark) and a TDF-100A enzymatic kit, respectively. The total protein level was measured by determining the nitrogen content using a Kjeltec 8,200 nitrogen analyzer (FOSS, Denmark), which was used to calculate the total protein content. Finally, the content of digestible carbohydrates was calculated using Formula (1). Three replicates were employed.


(1) $$\mathrm{Digestible carbohydrate content}=100-\left(\begin{array}{l} \%CL+\%TP+\\ \%DF+\%AS \end{array}\right)$$

#### 2.3.2 Mineral compositions

After LRO was digested and transferred into flasks, the compositions of minerals, including Ca, Cu, Fe, K, Na, Mg, Mn, P, Se, and Zn, were analyzed using inductively coupled plasma–mass spectrometer (ICP-MS) (Agilent 7,900, United States). The contents of each mineral are presented as mg/100 g fresh sample. Three replicates were used (12).

#### 2.3.3 Amino acid compositions

After digestion with 6 M HCl (10 mL) in a sealed ampoule at 110°C for 24 h, the supernatant of the LRO solution was transferred to a bottle to dry at 60°C. Then, the sample was dissolved in 0.2 M sodium citrate buffer (pH 2.2), and the supernatant was extracted and analyzed using a Biochrom 30^+^ Automatic Amino Acid Analyzer (Biochrom, England) with an ion-exchange column to measure the amino acid content. The amino acid content was calculated, and three replicates were employed (13, 14).

#### 2.3.4 Fatty acid compositions

LRO was mixed with an undecanoic acid (internal standard) methanol solution (2.0 mL) and pyrogallol (100 mg) in a tube. After heating at 80°C for 1 h, the sample was mixed with 15% boron trifluoride in methanol (7 mL) to convert fatty acids to esters at 80°C for 1 h. Then, hexane and saturated sodium chloride solution (15 mL) were added. The hexane layer was extracted and determined by autosampler gas chromatography (GC) with a flame ionization detector for the derivatized fatty acids, which were separated using an Agilent HP-88 column (60 mm × 0.25 mm, 0.2 μm). The temperatures were 270°C for the injector and 280°C for the detector. The oven temperature conditions were set as follows: the temperature was set to 100°C for 13 min, increased to 180°C (10°C/min) and held for 6 min, increased to 200°C (1°C/min) and held for 20 min, and finally increased to 230°C (4°C/min) and held for 10.5 min. Helium was used as the carrier gas. The fatty acids were calculated using fatty acid conversion factors from their methyl esters (12).

### 2.4 Phytochemical profile

#### 2.4.1 Total phenolic and flavonoid compounds

The content of total phenolic compounds (TPCs) in LRO harvested in the different seasons was determined using the Folin–Ciocalteu method, and the results are expressed as mg of gallic acid equivalent/100 g of fresh sample (mg GAE/100 g). The content of flavonoids (TF) in LRO was measured according to the aluminum chloride colorimetric method, and the result was expressed as mg of quecertin/100 g of fresh sample (mg QE/100 g) (3). The absorbance was measured at a wavelength of 760 nm to determine the TPC and 510 nm to measure the TF. Three replicates were employed.

#### 2.4.2 Quantification of phytochemicals via UPLC

To investigate how the main compounds in LRO changed during the different growth periods, the contents of eight compounds, namely, hyperoside, emodin, rhein, aloe-emodin, myricitrin, kaempferol, and isoorientin, were determined using an Agilent Technologies HPLC apparatus (1,290 Infinity II, Agilent, United States). Before the test, LRO (3 g) was added to methanol (50 mL) and then ultrasonicated for 30 min. After the mixture was filtered through a 0.22 μm filter, the supernatant was used as the final solution for quantitative analysis, which was based on the retention times and UV spectra of commercial compounds. The solvent system was composed of 0.1% formic acid solution (A) and acetonitrile (B). The following gradient elution method was applied: 0–10 min 95–82% A; 10–40 min 92–35% A; 40–53 min 35–10% A; and 53–55 min 90–95% A with a flow rate of 0.8 mL/min. The total run time and UV detector were set to 55 min and 280 nm, respectively. Symmetry (C_18_, 4.6 mm × 150 mm, 5 μm, Waters, Ireland) was maintained at ambient temperature (30.0°C).

### 2.5 *In vitro* antioxidant activity

The *in vitro* antioxidant activity of LRO was investigated according to a previously described method (3). For the DPPH radical scavenging activity assay, LRO (50–1,000 μg/mL) was added to 96-well plates with a methanol solution of DPPH (100 μL, 0.2 mM) and then incubated at 37°C for 30 min in the dark. The absorbance at 517 nm was measured using a Multiskan Go Microplate Spectrophotometer (Thermo Scientific, United States). To evaluate the superoxide radical scavenging activity of LRO, 100 μL of NADH-2Na (557 μM), 50 μL of PMS (45 μM) and 50 μL of NBT (108 μM) were mixed with LRO and then incubated at 25°C for 5 min. The absorbance was measured at 510 nm. To determine the reducing powder, LRO (100 μL) was added to a tube with sodium phosphate buffer (250 μL, pH 6.6) and 1% potassium ferrocyanide (250 μL), and then the solutions were incubated for 20 min at 50°C. After a 10% TCA solution (250 μL) was added, the sample was centrifuged at 4,000 rpm for 10 min, and the supernatants (50 μL) were removed and placed into a 96-well plate with an equal volume of distilled water and ferric chloride. The absorbance was measured at 700 nm.

In the above assays, distilled water and ascorbic acid (Vc) were used as negative and positive controls, respectively, for comparison. Then, to evaluate the antioxidant activity of the samples, the median scavenging concentrations (SC_50,_ the concentration of samples necessary to scavenge 50% of DPPH) were calculated using GraphPad Prism 8.0.2. Three replicates were employed.

### 2.6 Cellular antioxidant activity

In this test, after incubating the primary mouse macrophage RAW 264.7 cells (5 × 10^4^ cells/well) in medium prepared with 10% FBS and 90% DMEM under a humidified incubator of 5% CO_2_ at 37°C for 24 h, LRO (0.5–4 mg/mL, 10 μL) was added and incubated again for 24 h. Then, 10 μL of cell counting kit (ZETA Life, U.S.A.) was added and co-cultured for 30 min, the absorbance was measured at 450 nm. DMSO (0.1%) was used as a control.

For determining the cellular antioxidant activity, when the supernatants of RAW 264.7 cells (2 × 10^5^ cells/well) were discarded after incubating for 24 h, 2 mL of LRO (400 μg/mL) was added and cultured for 1 h. Dexamethasone (Dex,10 μg/mL) was used as the positive control. H_2_O_2_ (0.5 mM) was added to stimulate oxidant stress for 24 h. Finally, the activity of SOD (superoxide dismutase) in cells was detected using the assay kits (Solarbio, China) (3).

### 2.7 Statistical analysis

In this study, the results are expressed as the mean ± standard deviation. The contents of each mineral, amino acid, fatty acid, and compound in each sample were expressed as mg/100 g fresh weight, which were calculated according to the loss of mass during the drying process. The significant differences between two groups were analyzed using unpaired t tests. The data were analyzed by one-way ANOVA followed by Dunnett’s test for three or more groups using GraphPad Prism 8.0.2.

## 3 Results and discussion

### 3.1 Proximate compositions

**Figure 1:**
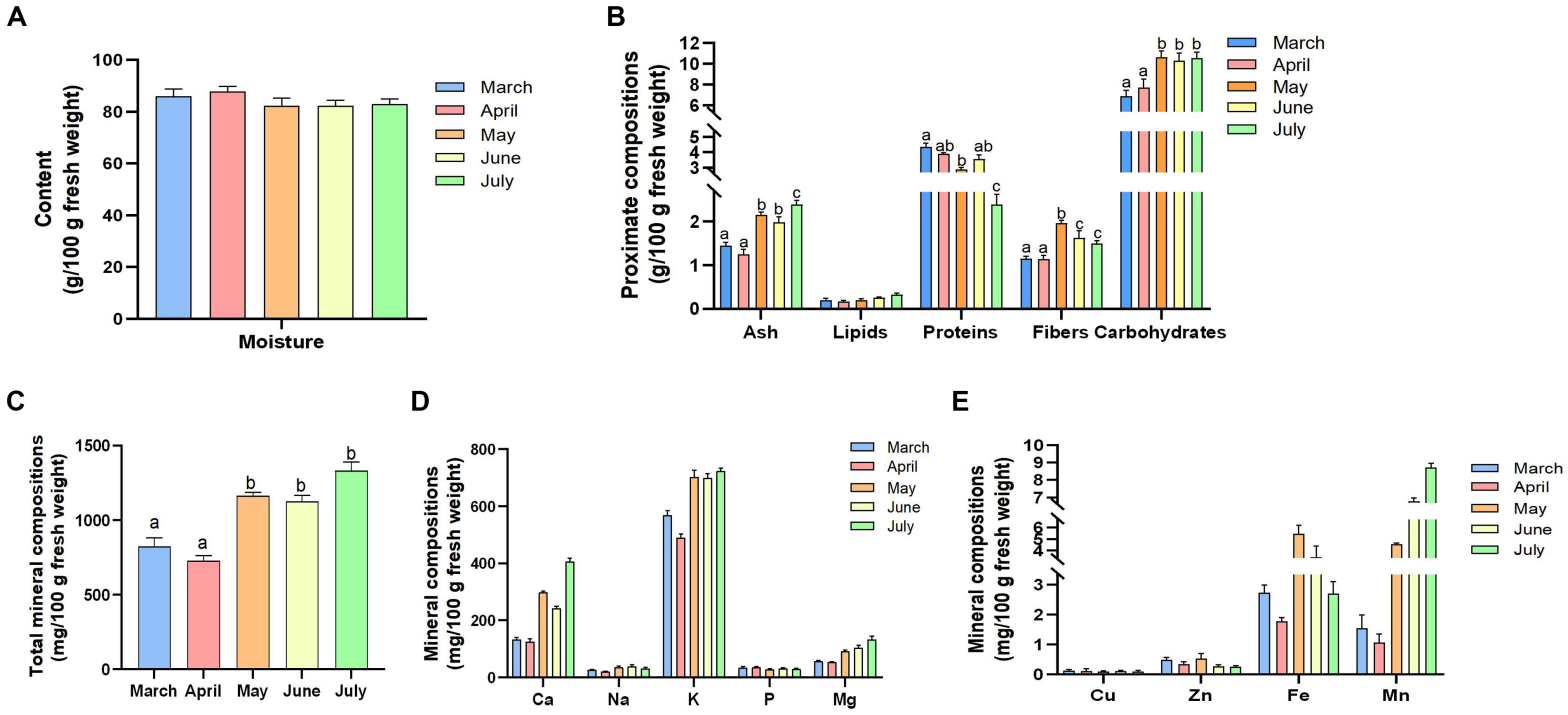
The contents of the proximate components [**(A)** moisture; **(B)** proximate compositions] and the mineral components [**(C)** total mineral compositions; **(D)** minerals of Ca, Na, K, Mg, and P; and **(E)**. other components of Cu, Zn, Fe, and Mn] of LRO during the different growth periods (the changes in the letters represent significant differences between the different groups, *p* < 0.05).

In this study, we first investigated the relationships between the proximate composition and harvest season of *R. officinale* leaf blades (LRO) and compared differences in macronutrients among various harvest stages. From Figure 1A, we can see that LRO contains a high moisture content (82 to 93%) during the harvest season; however, there is no significant difference from March to July (*p* > 0.05). Although the contents of macronutrients dynamically changed during the different growth periods, the most abundant component remained carbohydrates, followed by proteins, ash, fiber and lipids. Specifically, during the growth of this species from March to July, the protein content decreased significantly, and a significant difference in the protein content occurred between March and July (*p* < 0.05). The lipid content remained constant (*p* > 0.05); however, other components increased gradually (Figure 1B). The contents of ash, fiber and carbohydrates in May, June and July were greater than those in March and April (*p* > 0.05). As a result, fresh LRO harvested in May, which contains a relatively high fiber content, is widely used as a feed additive for humans and animals to improve digestion in some regions (15).

### 3.2 Mineral compositions

Vegetables are a significant source of minerals needed by humans, and the daily intake of K, Ca and Mg provides important cardiovascular and bone benefits (13, 14, 16). As shown in Figure 1C, the total content of minerals in the LRO changed dynamically during the different growth periods. The total content reached 1233.62 mg/100 g in July, followed by May and June. The ash content in LRO, which contains an abundance of inorganic salt and minerals, tended to change. Among these minerals, the content of K was the highest, with 723.17 mg/100 g fresh LRO in July.

According to the New Zealand Nutrition Database, the standard unit for a cup size of rhubarb is 265 g, and the total contents of Ca are 0.80 g/serving and 1.08 g/serving, which are lower than those in trim milk (1.67 g/serving) and standard milk (1.65 g/serving), respectively. The results showed that LRO was rich in Ca, as fresh LRO contained 405.74 mg/100 g Ca in July and 298.10 mg/100 g Ca in May (Figure 1D). Considering that the fresh samples have high moisture levels, the dry LRO contains abundant levels of *Ca.* In addition, Mg and P were detected; Ca, K, Mg, etc., may be antioxidant minerals and can be used to prevent oxidative stress. The contents of K, Ca and Mg in May, June and July were greater than those in March and April (*p* > 0.05) (Figure 1E). Due to potential health problems, a reduced intake of Na (less than 2 g/day in adults) has been recommended by the WHO (17). The Na content in LRO was low (20.29–39.14 mg/100 g) during the different harvest seasons. There was a significant difference in the Na content between March and May (*p* < 0.05).

The above results indicated that LRO could be employed as a good food supplement or feed additive to improve health and other metabolic processes, as LRO contains high levels of K and Ca but low levels of Na. Moreover, some trace minerals were detected, such as Cu, Zn, Fe and Mn, which play important roles in improving body functions. However, Se was not detected in LRO.

### 3.3 Amino acid compositions

Good proteins are necessary to maintain balance in the diet and provide the nutrients needed by the body. In our test, 17 amino acids were detected in fresh LRO. The results showed that the contents of amino acids decreased significantly from March to July, similar to the change in proteins. The total contents of amino acids determined varied in the following order: March (4158.17 mg/100 g) > April (3729.13 mg/100 g) > June (3453.45 mg/100 g) > May (2809.59 mg/100 g) > July (2261.55 mg/100 g) (Table 1). There were significant differences among the months (*p* < 0.05).

**Table 1:** The contents of amino acids in LRO during the different growth periods.

Amino acids	Mg/100 g fresh weight basis				
	March	April	May	June	July
Gly	507.16 ± 10.10^a^	808.83 ± 8.76^b^	614.04 ± 5.98^c^	855.39 ± 4.65^b^	503.33 ± 10.12^a^
Ala	232.57 ± 3.89^a^	170.28 ± 3.42^b^	119.60 ± 2.37^c^	125.74 ± 6.54^c^	50.46 ± 5.32^d^
Val*	254.98 ± 10.12^a^	201.50 ± 7.54^b^	148.16 ± 2.14^b^	143.45 ± 6.38^b^	114.70 ± 7.82^c^
Leu*	365.661 ± 8.63^a^	246.91 ± 8.98^b^	180.29 ± 5.87^c^	178.87 ± 5.43^c^	114.70 ± 3.48^d^
Ile*	189.14 ± 2.76^a^	131.97 ± 4.85^b^	105.32 ± 4.32^bc^	93.86 ± 2.76^d^	70.19 ± 2.38^de^
Pro	126.09 ± 3.27^a^	122.03 ± 4.13^a^	69.62 ± 1.36^b^	58.44 ± 2.54^b^	32.53 ± 2.19^c^
Ser	196.14 ± 3.48^a^	180.21 ± 4.51^a^	123.17 ± 4.67^b^	134.60 ± 5.42^b^	89.02 ± 3.21^c^
Cys	39.23 ± 3.12^a^	61.02 ± 2.64^b^	37.49 ± 1.42^a^	70.84 ± 1.8^8c^	51.36 ± 3.12^d^
Met*	25.22 ± 1.03^a^	11.35 ± 1.21^b^	7.14 ± 0.42^bc^	5.31 ± 0.32^c^	5.14 ± 0.26^c^
Thr*	186.33 ± 3.67^a^	146.16 ± 4.66^b^	103.53 ± 2.38^c^	111.47 ± 5.36^bc^	85.6 ± 2.38^d^
Phe*	322.23 ± 6.54^a^	263.93 ± 6.88^b^	219.56 ± 8.34^c^	255.02 ± 7.10^b^	140.38 ± 5.32^d^
Tyr	168.12 ± 4.81^a^	129.13 ± 5.34^b^	87.47 ± 3.43^c^	100.95 ± 4.23^bc^	63.34 ± 2.31^d^
Asp	353.05 ± 3.26^a^	263.93 ± 2.99^b^	182.07 ± 5.66^c^	216.06 ± 6.32^c^	135.25 ± 4.23^d^
Glu	507.16 ± 4.12^a^	808.83 ± 4.67^b^	614.04 ± 3.89^c^	855.39 ± 4.44^b^	503.33 ± 3.28^a^
Lys*	386.68 ± 5.77^a^	397.32 ± 2.36^a^	315.95 ± 2.13^b^	540.16 ± 2.73^c^	393.76 ± 3.88^a^
Arg	141.50 ± 2.19^ab^	126.29 ± 4.12^a^	153.51 ± 4.82^ab^	175.33 ± 2.25^b^	140.38 ± 3.16^ab^
His*	404.89 ± 6.27^a^	297.99 ± 7.99^b^	217.77 ± 10.12^c^	246.17 ± 8.23^bc^	154.08 ± 6.54^d^
Total	4158.17 ± 85.35^a^	3729.13 ± 30.51^b^	2809.59 ± 21.20^c^	3453.45 ± 36.14^d^	2261.55 ± 33.25^e^
E-Total	2174.35 ± 70.21^a^	1758.14 ± 25.23^b^	1335.18 ± 15.21^c^	1645.16 ± 31.45^d^	1129.92 ± 28.62^e^

A change in the letter represents a significant difference between the different groups, *p* < 0.05.

Among these amino acids, Glu and Gly were most abundant; Glu and Gly play important roles in the physiological activity of the body and can improve the flavor of LRO. In addition, eight essential amino acids that cannot be synthesized by the body were detected in fresh LRO in March at 2174.35 mg/100 g.

In addition, the content of essential amino acids was greatest in March, followed by April (1758.14 mg/100 g), June (1645.16 mg/100 g), May (1335.18 mg/100 g) and July (1129.92 mg/100 g). Moreover, LRO contains hydrophobic amino acids and sulfur-containing amino acids (Table 1), which were reported to exhibit antioxidant activity (18). This result indicated that LRO is a high-quality protein and may be a useful food additive to protect the body by improving immune functions.

### 3.4 Fatty acid compositions

Fatty acids are fundamental structural components and participate in important metabolic pathways to resist biotic and abiotic stresses (13, 14). However, fatty acids, especially short-chain fatty acids (*n < 6*), are sensitive to temperature; under high temperatures, the double bonds in polyunsaturated fatty acids are more vulnerable to attack by free radicals (19). Compared with previous studies in which a sample of this species was dried under shade (3), the samples dried in an oven at 60°C contained significantly less fatty acids, and short-chain fatty acids (*n* < 10) were not detected. In addition, unlike the total protein content, the total fatty acid content (287.01 mg/100 g in fresh LRO in March) did not change significantly during the different growth periods (Table 2). Except for the significant change in fatty acid content between March and May (*p* < 0.05), there were no significant differences among months (*p* > 0.05).

**Table 2:** The fatty acid contents of LRO during the different growth periods.

Fatty acids	LRO (mg/100 g fresh weight basis)				
	March	April	May	June	July
C10	2.56 ± 0.14^a^	0.96 ± 0.10^b^	--	1.77 ± 0.21^c^	1.63 ± 0.06^c^
C12	3.36 ± 0.34^ac^	3.70 ± 0.35^a^	3.80 ± 0.52^a^	5.49 ± 0.87^b^	4.06 ± 1.00^cd^
C13	2.79 ± 0.54^a^	3.31 ± 0.43^ac^	3.50 ± 0.35^bc^	4.78 ± 0.34^d^	3.44 ± 0.4^bc^
C14	1.63 ± 0.14^a^	1.45 ± 0.09^a^	3.03 ± 0.19^b^	2.73 ± 0.12^b^	3.27 ± 0.65^b^
C14:1	0.92 ± 0.06^a^	0.87 ± 0.03^a^	1.50 ± 0.43^b^	1.30 ± 0.03^ab^	1.21 ± 0.23^ab^
C15	2.42 ± 0.53^a^	3.20 ± 0.12^b^	3.09 ± 0.05^b^	4.32 ± 0.06^c^	3.15 ± 0.12^b^
C15:1	78.04 ± 4.56^a^	62.58 ± 3.21^b^	66.22 ± 1.15^b^	66.94 ± 2.01^b^	67.62 ± 0.99^b^
C16	–	–	1.10 ± 0.01	–	–
C16:1	2.40 ± 0.03^a^	0.69 ± 0.05^b^	2.11 ± 0.11^a^	1.06 ± 0.07^bc^	1.20 ± 0.02^bc^
C17	2.55 ± 0.21^a^	2.74 ± 0.42^a^	2.84 ± 0.15^a^	3.98 ± 0.45^b^	3.08 ± 0.55^ab^
C17:1	5.84 ± 0.47^a^	7.75 ± 0.65^b^	1.01 ± 0.02^a^	10.38 ± 0.76^d^	9.28 ± 1.01^d^
C18	–	–	1.15 ± 0.03	–	–
C18:1	33.01 ± 1.01^a^	12.63 ± 1.34^b^	23.03 ± 0.89^c^	33.38 ± 2.21^a^	37.90 ± 3.21^a^
C18:2	–	0.66 ± 0.02^a^	1.35 ± 0.32^b^	–	49.62 ± 4.52^c^
C18:3	143.32 ± 4.59^a^	146.58 ± 3.98^a^	94.61 ± 2.56^b^	116.18 ± 4.63^bc^	53.07 ± 3.98^d^
C20	1.40 ± 0.21	–	–	–	–
C20:1	3.11 ± 0.65	–	–	–	–
C20:4	–	–	–	–	2.60 ± 0.54
C21	2.62 ± 0.76^a^	3.89 ± 0.34^b^	3.43 ± 0.38^b^	3.83 ± 0.32^b^	2.62 ± 0.32^a^
C21:1	1.03 ± 0.03^a^	2.40 ± 0.05^b^	1.37 ± 0.02^a^	1.44 ± 0.21^a^	1.38 ± 0.04^a^
C23	–	2.16 ± 0.01^a^	–	3.21 ± 0.03^b^	–
C24:1	–	–	–	–	1.17 ± 0.02
Total	287.01 ± 17.79^a^	255.55 ± 7.23^abc^	213.13 ± 18.90^b^	260.80 ± 22.03^c^	246.31 ± 11.93^abc^
SFA	19.33 ± 3.78^a^	21.39 ± 2.98^a^	21.94 ± 3.31^a^	30.11 ± 4.23^b^	21.25 ± 3.24^a^
MUFA	124.35 ± 6.5^a^	86.91 ± 10.82^b^	95.24 ± 14.05^ab^	114.51 ± 10.15^ab^	119.77 ± 13.01^a^
PUFA	143.32 ± 11.02^a^	147.24 ± 11.79^a^	95.96 ± 14.00^b^	116.18 ± 6.51^b^	105.30 ± 5.51^b^

A change in the letter represents a significant difference between the different groups, *p* < 0.05.

Essential fatty acids are necessary for the human diet and food. In this experiment, 124.35 mg/100 g of monounsaturated fatty acids (MUFAs) and 143.32 mg/100 g of polyunsaturated fatty acids (PUFAs) were found in the fresh LRO in March. The contents of PUFAs in March and April were greater than those in May, June and July (*p* < 0.05). The most abundant fatty acid was C18:3, with contents of 20.11 mg/100 g and 143.32 mg/100 g; this fatty acid played important roles in maintaining the high quality of the food (13, 14). Eicosapentaenoic acid and docosahexaenoic acid have cardiovascular protective effects and anti-inflammatory and antioxidant activities; therefore, as LRO contains essential fatty acids, the leaves have nutrient qualities and could be used as a functional food to prevent some chronic cardiovascular diseases.

### 3.5 Total phenolic and flavonoid compounds

**Figure 2:**
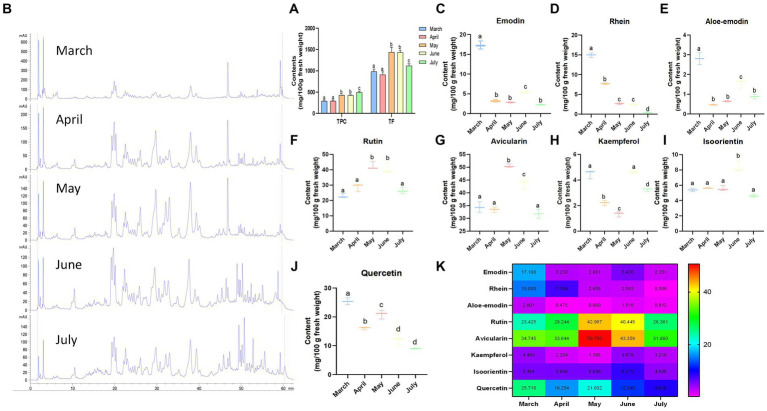
The contents of phenolic compounds and flavonoids in LRO **(A)** UPLC chromatograms at the different harvest seasons for determining eight compounds **(B)**; the contents of emodin [**(C)**, Rt 59.274 min], rhein [**(D)**, Rt 57.994 min], aloe-emodin [**(E)**, Rt 53.827 min], rutin [**(F)**, Rt 33.059 min], avicularin [**(G)**, Rt 38.090 min], kaempferol [**(H)**, Rt 49.557 min], isoorientin [**(I)**, Rt 25.004 min], and quercetin [**(J)**, Rt 46.767 min]; and the heatmap for eight compounds in LRO **(K)** (the change in letter represents the significant difference between the different groups, *p* < 0.05).

Selecting the most bioactive ingredients for functional food production is very important, and phenolic compounds and flavonoids may be the active components of rhubarb (11). Hence, the TPC and TF values of fresh LRO were evaluated. The differences were significant during the different harvest periods (*p* < 0.05). For TPC (unlike proteins), the TPC of LRO increased gradually as growth progressed and reached a maximum in July (Figure 2A). The contents were 294.04 mg GAE/100 g in fresh LRO in March, 290.30 mg in April, 430.45 mg in May, 425.57 mg in June, and 497.11 mg in July. In addition, similar trends were observed for TF, in which the contents varied in the following order: June (1435.27 mg QE/100 g) > May (1320.45 mg/100 g) > July (1118.36 mg/100 g) > March (985.63 mg/100 g) > April (913.77 mg/100 g) (Figure 2A) This finding is consistent with the carbohydrate content in LRO.

### 3.6 Quantification of phytochemicals using UPLC

The multiple health benefits of traditional plants are derived from their abundance of bioactive compounds and metabolites (20). To further demonstrate the above results, UPLC was used to determine the contents of eight chemicals in LRO, and the results revealed two kinds of compounds, quinones and flavonoids. The UPLC chromatograms are presented in Figure 2B, and the compounds were detected at different retention times (Rt). For isoorientin, the Rt was 25.004 min, followed by 33.059 min for rutin, 38.090 min for avicularin, 46.767 min for quercetin, 49.557 min for kaempferol, 53.827 min for aloe-emodin, 57.994 min for rhein and 59.274 min for emodin. These compounds were also detected by Xian (21).

Anthraquinones are important compounds from the genus *Rheum*. Chrysophanol, physcion, emodin, aloe-emodin, and citreorosein were identified from the aerial parts of *R. officinale* by Xian (21). As shown in Figures 2C–E, the contents of anthraquinones, including emodin, rhein, and aloe-emodin, reached a maximum in March, and the contents were 17.16, 15.05 and 2.80 mg/100 g, respectively, in fresh LRO. However, with increasing harvest time, the content of this compound rapidly decreased, with values of 2.35, 0.31 and 0.81 mg/100 g in July. This result is similar to those in a paper published by Yao and Liu (22) and Li et al. (23), who reported that the total content of anthraquinones was greater in *Polygonum cuspidatum* and *P. multiflorum* plants harvested in spring than those harvested in autumn. Specifically, the contents of physcion-8-O-β-D-glucoside and physcion-8-O-(6′-methylmalonyl)-glucopyranoside decreased as the seasons progressed, while those of the other seven anthraquinones (including emodin-1-O-glucoside, emodin, emodin-6-O-glucoside, 1-methylemodin, emodin-8-O-β-D-glucoside, emodin-8-O-(6′-methylmalonyl)-glucopyranoside and physcion) increased in spring and summer but decreased sharply in autumn (23). The level of anthraquinones decreased because the chemical properties of anthraquinones are unstable, especially at high temperatures. Anthraquinones in the LRO may be degraded as the temperature gradually increases from spring to autumn.

Subsequently, the contents of five flavonoids (Figures 2F–J), including rutin, avicularin, kaempferol, quercetin and isoorientin, in LRO were determined using UPLC. Avicularin and isoorientin were identified from this species for the first time. Among these compounds, the content of avicularin was the highest, followed by that of rutin and quercetin. In comparison, LRO contains less kaempferol and isoorientin. For the different harvest times, the content of avicularin in LRO reached a maximum in May (50.70 mg/100 g), followed by June (40.36 mg/100 g); a similar trend was observed for rutin, and LRO harvested in May contained the highest amount of this compound (42.97 mg/100 g). However, the highest level of quercetin in LRO was found in March (25.72 mg/100 g), followed by May (21.60 mg/100 g). In addition, the levels of kaempferol and isoorientin reached a maximum in June, at 4.68 and 8.27 mg/100 g, respectively. Zhang et al. (24) reported that the flavonoid content of young red leaves in summer was 2-fold greater than that in winter, and the greatest flavonoid content in gingko leaves was found in summer (25). The flavonoid content in *Berberis thunbergii* was also high in summer, while the flavonoid content in *B. thunbergii* var. *atropurpurea* was high in winter (26). Studies have shown that the biosynthesis of plant flavonoids is often affected by environmental factors, such as ultraviolet radiation, and plants tend to synthesize more flavonoids in response to UV stress (27, 28). Considering that the intensity of UV radiation in Gansu Province collected by LRO gradually increased from March to July, the flavonoid content was greater in May and June (Figure 2K). Hence, the levels of flavonoids were strongly affected by harvest time and species.

### 3.7 *In vitro* antioxidant activity

**Figure 3:**
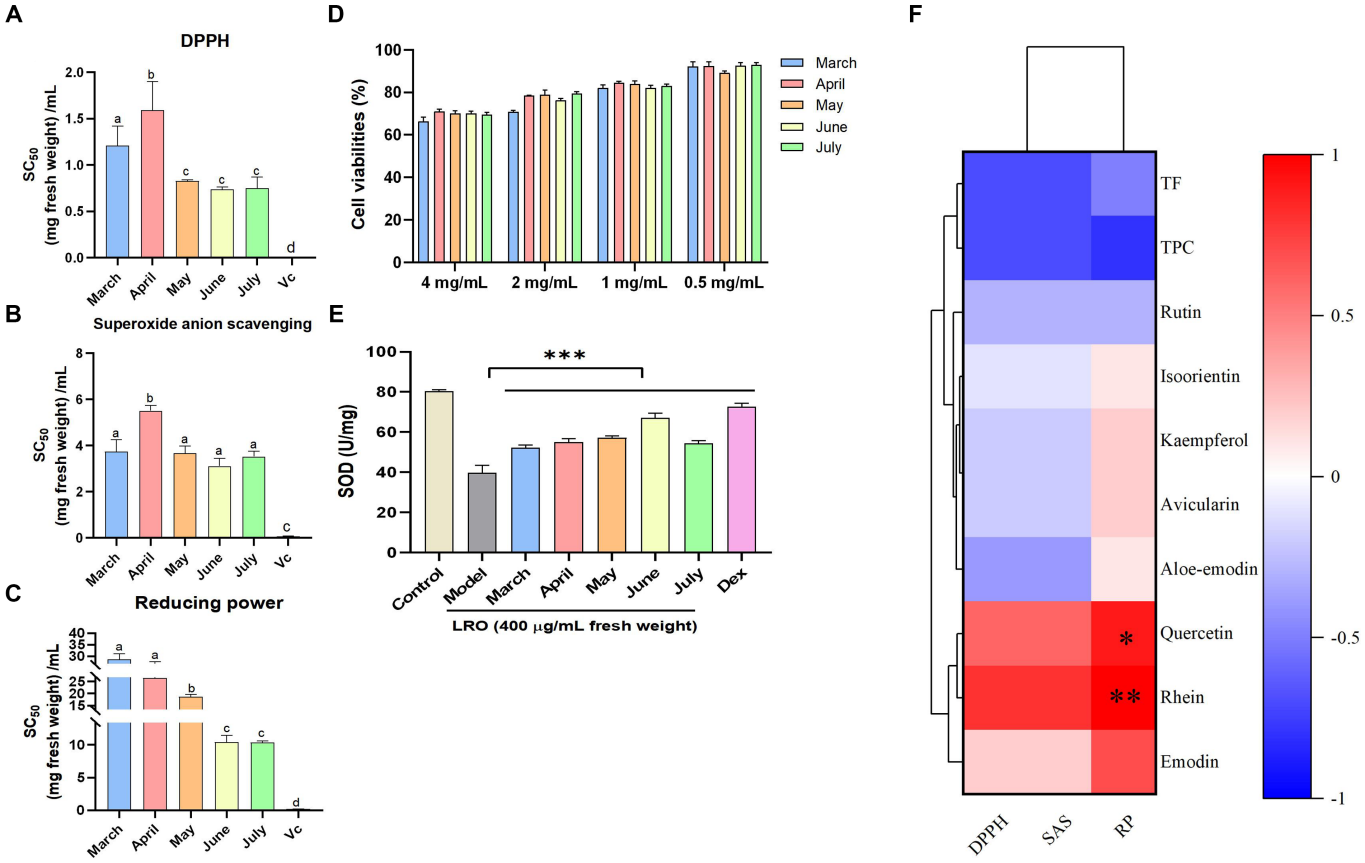
The *in vitro* antioxidant activity of the DPPH test **(A)**, superoxide anion scavenging test **(B)** and reducing power test **(C)**; the cytotoxicity of LRO **(D)**, and the SOD activity in RAW264.7 cells induced by H2O2 **(E)**, Heatmap of Spearman’s correlation between chemical constituents and the bioactivities **(F)** [**(D)** DPPH; **(E)** superoxide anion scavenging (SAS); **(F)** reducing power (RP)] of LRO (the change in letter represents the significant difference between the different groups in vitro antioxidant activity, *p* < 0.05; *represents the significant difference between the different groups in the heatmap).

A previous report showed that *R. officinale* stalks and stems exhibited antioxidant and anti-inflammatory activities (3). In this study, the antioxidant activity of fresh LRO at different harvest times was evaluated and found to be related to metabolite content changes. According to the DPPH test, the LRO harvested in June presented the greatest antioxidant activity, and the SC_50_ value was 0.74 mg fresh weight/mL; the LRO collected in April exhibited the worst activity, with an SC_50_ value of 1.59 mg fresh weight/mL (Figure 3A). A similar trend was observed for antioxidant activity in the superoxide radical scavenging test. LRO harvested in June had the greatest activity, which increased in a dose-dependent manner, and the SC_50_ value was 3.11 mg fresh weight/mL (Figure 3B).

In addition, the reducing power of LRO based on an electron transfer reaction was evaluated, and LRO harvested in June and July presented the greatest activity, with SC_50_ values of 10.44 and 10.35 mg fresh weight/mL, respectively. The concentration of the positive control Vc was 0.21 mg/mL (Figure 3C).

### 3.8 Cellular antioxidant activity

In cytotoxicity test, result showed that at the concentrations of 0.4–4 mg/mL, LRO is safe *in vitro*. Even at 4 mg/mL, the cell viabilities of LRO harvested at the different time were more than 70% (Figure 3D). SOD provide first-line cellular protection contributing to prevent cellular damage and maintain a balance between free radical production and oxidative stress after stimulated by H_2_O_2._ Result showed after the treatment of LRO, the activity of SOD in RAW 264.7 cells was significantly activated compared with the model group in a dose-dependent manner (*p < 0.01*). at the concentrations of 400 μg/mL, LRO harvested in June presented the greatest activity with the SOD activity of 67.08 U/mg Prot (*p* < 0.001), next were May, April, July and March (Figure 3E). This result is similar to the result of previous study, and proved that LRO harvested in June had the greatest antioxidant activity.

### 3.9 Correlations between phytochemicals and antioxidant activity potentials

The above results showed that LRO exhibits radical scavenging activity in the DPPH assay, reducing power test and superoxide radical scavenging test; therefore, LRO could alleviate the cellular damage induced by superoxide radicals and help prevent aging and some degenerative diseases. Considering that the fresh LRO harvested in May and June contained the most flavonoids, there was a direct correlation between the flavonoid content of LRO and its antioxidant activity, and the antioxidant potential was greatly affected by harvest time (3, 29).

Spearman’s analysis was used to determine the correlation between TFC, TPC, eight phytochemical contents, and the SC_50_ values determined by DPPH, superoxide radical scavenging, and reducing power tests. As shown in Figure 3F, there was a strong negative correlation between TF and TPC and DPPH (*r* = −0.7; −0.7), ABTS (*r* = −0.7; −0.7), and FRAP values (*r* = −0.5; −0.8). These results showed that the TF and TPC in LRO, which were very abundant, presented strong antioxidant activity. A negative correlation with antioxidant activity was also observed among the five flavonoids except quercetin. These results showed that flavonoids were important contributors to the antioxidant activity of LRO. Our previous study also revealed that *R. officinale* leaves exhibit radical scavenging activity, and rutin, avicularin, kaempferol, quercetin and isoorientin may be active compounds (3). The antioxidant activity of these compounds has also been reported by many international groups (30–32). However, two quinones, emodin and rhein, are positively correlated with this activity, and this result showed that quinones are not the main compounds that generate the antioxidant activity of LRO. This conclusion is consistent with the previous test in which the high contents of quinones in LRO exhibited weak antioxidant activity in March.

## 4 Conclusion

The applications and biological activities of foods or plants and their components depend on their nutrients and chemical compounds, which are affected by the different harvest seasons. Reports have demonstrated that polyphenols in the edible stalks of rhubarb are affected by harvest date (33). As above described, the fresh aerial parts of *R. officinale*, especially the leaves contained the abundant of nutrients, are common eaten as vegetables by local people and animals in these regions. However, it is unclear which season is more suitable for harvesting the leaf blades of *R. officinale* (LRO), and the relationships among of the content of nutrients and chemicals, antioxidant activity and harvest season should be investigated comprehensively.

Considering that the leaves have the potential to be functional food or sources of nutrients in the food industry, we studied the changes in nutrients, phytochemical profiles and antioxidant activity of LRO during different growth periods, which will lay the foundation to choose more suitable harvest time. Result showed that the nutrient content of LRO is high and that the plants are rich in fiber and proteins and minerals (K, Ca, Mg.), essential amino acids and essential fatty acids. Moreover, LRO contains flavonoids, terpenoids, quinones and other secondary metabolites, which contribute to its antioxidant activity. However, the nutrients, phytochemical profile and antioxidant activity of LRO changed dynamically during the different growth periods. The protein content gradually decreased from March to July, which was confirmed by amino acid analysis. The ash and carbohydrate contents of LRO increased with increasing harvest time, and the contents reached a maximum in May, followed by July and June. This trend was also observed in the phytochemical analysis. The contents of total phenolic and flavonoid compounds were also the highest in May or June, which contributed to the antioxidant activity of LRO *in vitro*. However, with increasing harvest time, the anthraquinone content rapidly decreased, possibly due to the instability of the chemical properties at high temperatures. The above results showed that rhubarb leaves are a source of nutrients and chemical compounds (flavonoids, etc.) with antioxidant activity and can be used to produce functional foods. Therefore, the harvest date, which largely determines the property of LRO, should be considered when selecting the raw material for production.

However, some limitations of this study are still existed. Firstly, although the changes of the total phenolic and flavonoid compounds as active components collected from the different harvest time has been determined, more secondary metabolites appeared in UPLC chromatograms have not been explained except eight certain compounds. High-quality qualitative and quantitative metabolomics must be studied to identify more chemicals further. Secondly, while harvesting time is an important factor affecting the chemical composition, it is crucial to consider other parameters such as irrigation, weather, temperature and soil condition that could also play a significant role. Rocchetti et al. (34) proved that terroir, cultivar., seasonality, and farming systems were found to significantly shape the metabolomic profile of tomato fruits and, consequently, their antioxidant and enzyme inhibition capacities. The high temperatures will affect the stable of anthraquinones, and results in the decrease of this kind of compounds. Moreover, ultraviolet radiation will affect the biosynthesis of flavonoids, and more flavonoids would be produced in response to UV stress (27, 28). In this test, to avoid excessive external factors affecting the test results, we collected the samples in the same soil with the same weather and irrigation. However, the intrinic interaction between other additional factors and the chemical composition of rhubarb are still unrevealed and should be solved in the future.

In summary, this study indicated that the leaf blades of *R. officinale* are a good source of nutrients and chemical components and can be used for functional food production. The nutrients and chemical components related to the antioxidant activity of LRO changed according to the harvest season.

## Data availability statement

The original contributions presented in the study are included in the article/Supplementary material, further inquiries can be directed to the corresponding authors.

## Author contributions

LD: Writing – original draft. XM: Writing – original draft. YM: Writing – review & editing. XY: Writing – original draft. BL: Writing – original draft. JH: Writing – original draft. YW: Writing – original draft. PD: Writing – original draft. JZ: Writing – review & editing. XS: Conceptualization, Funding acquisition, Supervision, Writing – original draft, Writing – review & editing.
